# A Medical Legend

**Published:** 1875-07

**Authors:** 


(Apropos to the matter of diagnostic errors, we have received the following poem from its author, who asks that he be allowed to remain incognito.—Editor.)

*A MEDICAL LEGEND.*

On the banks of Hudson River
  There dwelt a High Dutch doctor,
Of home-made potions, powders, pills,
  A famous old concoctor.

To him the sick from far and near
  In daily crowds did come,
And by his skill and home-made draughts
  He made a pretty plum.

One day a message came in haste,
  To visit Nancy Bell,
Who did not waste away at all.
  And yet was far from well.

She was a maiden of eighteen,
  Of figure lithe and tall,
In great request by all the beaux
  At every country ball.

Not only was her figure fine,
  But she was wondrous fair,
And in the mazes of the dance
  Her grace was something rare.

But now she drooped and moped all day,
  Although her bodily vigor
Did not perceptibly decrease,
  Per contra, she grew bigger.

The doctor felt his patient's pulse,
  And looked upon her tongue,
And felt great interest in her case,
  She was so fair and young.

And when her anxious parents plied
  Their questions on the case,
The learned doctor answered them
  With wise and serious face:

"Pray have no fear," he mildly said,
  "Nor let the name alarm you;
'Tis dropsy, which I can remove
  So quickly it will charm you.

"I am no specialist like some,
Who, being narrow-minded,
Profess great skill in little things,
By which folks oft are blinded.

"But tho' supreme o'er all complaints,
I rule with skill that none fears,
Yet when it comes to dropsy, then
I distance all my compeers.

"Some doctors tap, they know no better;
'Tis a coarse and barbarous notion;
I scorn such rude mechanic art,
With my unfailing potion.

"Just let your daughter duly take
The medicine I shall send,
And then you need retain no fear,
But she will quickly mend."

But though the medicine she took,
She grew no better fast,
And faith in doctor's promises
Began to fail at last.

At length one night the crisis came;
Nancy was awful sick,
But in the morning she was well—
They called that dropsy DICK.

